# No association between polymorphisms/haplotypes of the vascular endothelial growth factor gene and preeclampsia

**DOI:** 10.1186/1471-2393-11-35

**Published:** 2011-05-16

**Authors:** Idalia Garza-Veloz, Claudia Castruita-De la Rosa, Raul Cortes-Flores, Victoria Martinez-Gaytan, Jose E Rivera-Muñoz, Elda A Garcia-Mayorga, Esteban Meza-Lamas, Augusto Rojas-Martinez, Rocio Ortiz-Lopez, Margarita L Martinez-Fierro

**Affiliations:** 1 Laboratorio de Medicina Molecular, Unidad Academica de Medicina Humana y Ciencias de la Salud, Universidad Autonoma de Zacatecas, Carretera Zacatecas-Guadalajara Km.6. Ejido la Escondida, Zacatecas, Mexico; 2 Unidad Medica Materno-Fetal del Noreste, Unidad Medica de Alta Especialidad No. 23 Instituto Mexicano del Seguro Social, Avenida Constitucion y Felix U. Gomez, Col. Centro, Monterrey, Mexico; 3 Unidad Academica de Enfermeria, Universidad Autonoma de Zacatecas, Carretera Zacatecas-Guadalajara Km.6. Ejido la Escondida, Zacatecas, Mexico; 4 Department of Biochemistry and Molecular Medicine, University Hospital Universidad Autonoma de Nuevo Leon, Av. F. I. Madero S/N, Col. Mitras Centro, Monterrey, Mexico; 5 Centro de Investigacion y Desarrollo en Ciencias de la Salud, Universidad Autonoma de Nuevo Leon, Calle Carlos Canseco S/N, Col. Mitras Centro, Monterrey, Mexico

## Abstract

**Background:**

Preeclampsia (PE) is the first worldwide cause of death in pregnant women, intra-uterine growth retardation, and fetal prematurity. Some vascular endothelial grown factor gene (*VEGF*) polymorphisms have been associated to PE and other pregnancy disturbances. We evaluated the associations between *VEGF *genotypes/haplotypes and PE in Mexican women.

**Methods:**

164 pregnant women were enrolled in a case-control study (78 cases and 86 normotensive pregnant controls). The rs699947 (-2578C/A), rs1570360 (-1154G/A), rs2010963 (+405G/C), and rs25648 (-7C/T), *VEGF *variants were discriminated using Polymerase Chain Reaction - Restriction Fragment Length Polymorphism (PCR-RFLP) methods or Taqman single nucleotide polymorphism (SNP) assays.

**Results:**

The proportions of the minor allele for rs699947, rs1570360, rs2010963, and rs25648 *VEGF *SNPs were 0.33, 0.2, 0.39, and 0.17 in controls, and 0.39, 0.23, 0.41, and 0.15 in cases, respectively (*P *values > 0.05). The most frequent haplotypes of rs699947, rs1570360, rs2010963, and rs25648 *VEGF *SNPs, were C-G-C-C and C-G-G-C with frequencies of 0.39, 0.21 in cases and 0.37, 0.25 in controls, respectively (*P *values > 0.05)

**Conclusion:**

There was no evidence of an association between *VEGF *alleles, genotypes, or haplotypes frequencies and PE in our study.

## Background

Pre-eclampsia (PE) is a multi-organ disorder defined as elevated blood pressure (>140/90 mmHg) and proteinuria that may develop from 20 weeks gestation. PE is the first cause of maternal death in the western world and affects 5 to 8% of pregnant women, depending on the studied population and definitions of PE [1-3]. The Mexican Secretary of Health Registry reported 278 deaths by PE in 2007 in the country [4].

The etiopathology of PE remains unknown, but improper placentation has been suggested as a possible etiologic factor. This process involves the abnormal infiltration of the cytotrophoblast into the decidual endometrium and development of the spiral arteries originated from the myometrium. As a result, the diameter of the spiral arteries enlarge while arteries lose elasticity to allow an increased demand of blood flow to satisfy the intensifying demands of the growing fetus. Improper cytotrophoblast invasion may affect vascular remodelling and blood supply to the fetus, leading to local placental hypoxia [5-7]. Expression of vascular endothelial growth factor (*VEGF*) has been implied as a hypoxia-induced factor possibly associated to PE, as has been suggested previously [8]. Gu *et al*. observed increased mRNA and protein levels of *VEGF *in response to hypoxia in cultured cytotrophoblasts [9]. Zhou *et al*. found that cytotrophoblast differentiation and invasion during pregnancy is regulated through the *VEGF *receptor-2. They also found that *VEGF *regulates cytotrophoblast survival and that expression is deregulated in severe forms of PE.[10] Several authors reported an association between some *VEGF *polymorphisms and PE in selected populations, with some discrepancies [3,11-14]. No association studies for gene polymorphisms and PE have been reported for the Mexican population, despite the prevalence of this pregnancy disorder in the region. The purpose of the present study was to analyze the association of rs699947 (-2578C/A), rs1570360 (-1154G/A), rs2010963 (+405G/C), and rs25648 (-7C/T) *VEGF *polymorphisms in a sample of Mexican pregnant women enrolled during 2009-2010.

## Methods

### Patients and samples collection

This study was approved by the Institutional Review Board of the Unidad Medica de Alta Especialidad (UMAE) # 23 of the Instituto Mexicano del Seguro Social (IMSS) in Monterrey, Mexico. One hundred sixty-four unrelated pregnant women (86 PE cases and 78 normotensive controls), who provided written informed consent for their participation, were enrolled from the High Risk Pregnancy Outpatient Service of the UMAE # 23 between September 2009 and April 2010. Women between 15-38 years old with singleton pregnancy, 16-38 gestation weeks (GW), who lived in the metropolitan area of Monterrey, were eligible to participate. The PE diagnosis was made according to the guidelines of the International Society for the Study of Hypertension in Pregnancy [15]. In general, these guidelines include two recordings of diastolic blood pressure of 90 mmHg at least 4 h apart in previously normotensive women and proteinuria of 300 mg or more in 24 h, or two readings of at least ++ on dipstick analyses. PE was considered severe if blood pressure of 160 mm Hg systolic or higher or 110 mm Hg diastolic or higher on two occasions at least 6 hours apart while the patient was on bed rest, and proteinuria of 5 g or higher in a 24-hour urine specimen or 3+ or greater in two random urine samples collected at least 4 hours apart were present [16]. Exclusion criteria included multiple pregnancies, pregnancies with structural or chromosomic fetus malformations detected by ultrasound, chronic hypertension, chronic renal disease, gestational diabetes mellitus, and underlying medical diseases such as rheumatoid arthritis, and systemic lupus erythematosus.

A questionnaire, consisting of demographic and gynecologic data and risk factors (including family history of PE, cardiovascular disease, type 2 diabetes mellitus or autoimmune diseases) was administered to each patient by the participant gynecologist, and a venous blood sample was obtained using EDTA collection tubes. Blood samples were maintained at 4°C until their processing.

### SNP selection

Criteria for SNP selection included: SNPs for which an association with PE has been reported in other populations, and considering the potential impact on gene expression only SNPs located in promoter region of *VEGF *gene or inside its 5'UTR were considered. According to these criteria, rs699947 and rs1570360 variants (located in the *VEGF *promoter), and rs2010963 and rs25648 (located in 5'UTR of the *VEGF *gene) were included. Relative to translation start site, the variants rs699947 and rs1570360 are known as -2578C/A and -1154G/A, respectively [17,18]; while the rs2010963 SNP is called +405G/C (according to mRNA position) or -634G/C (relative to translation start site). In the same sense, rs25648 alternative names are 534C/T and -7C/T respectively.

### Nucleic acid extraction and genotyping

DNA was isolated from 1 mL of blood sample using a standard fenol/chloroform method coupled to ethanol precipitation. Table 1 shows a summary of the genotyping methods employed for the variants screening. PCR coupled to Restriction Fragment Length Polymorphism (PCR-RFLP) method was used to discriminate the rs699947, rs1570360, and rs2010963 *VEGF *polymorphic variants, respectively. We used the protocols described by Banyasz I *et al *(rs699947 and rs2010963) [3] and Liu Q *et al*. (rs1570360) [19], respectively. Some modifications in the primer sequences were included; these modifications are indicated in bold letters in Table 1. All the PCR reactions were made in a volume of 10 μl containing 100 ng of genomic DNA, 0.5 μM of each primer, and 1× of GoTaq Colorless Master Mix (Promega: Madison, WI). PCR cycling conditions were as follows: 94°C for 5 min; 35 cycles of 94°C for 30 s, 60°C of annealing temperature for 30 s, and 72°C for 30 s; a final extension step of 72°C for 5 min was included. PCR products were digested with *Bgl *II (rs699947), *Mnl *I (rs1570360), and *BsmF *I (rs2010963) at 37°C (rs699947 and rs1570360) or at 65°C (rs2010963) overnight, respectively. The rs699947 and rs2010963 digested products were electrophoresed through 3.5% agarose gels and visualized by ethidium bromide staining. Digested products of rs1570360 *VEGF *variant were visualized using the DNA 1000 LabChip kit on the Agilent 2100 Bioanalyzer (Agilent: Palo Alto, CA). Length of PCR and PCR-RFLPs products are shown in Table 1.

**Table 1:** Genotyping methods description.

SNP ID	Primer sequences*	Genotyping method (restriction endonuclease)	PCR- RFLP products length (bp)	Reference†
rs699947	Fw 5'-GCCTTAGGACACCATACC**GATG**-3' Rv 5'-**GC**TGCCCCAGGGAACAAAGT**TG**-3'	PCR/RFLP (*Bgl *II)	CC: 285 CA: 285,206,79 AA: 206,79	Modified from [3]
rs1570360	Fw 5'-TCCTGCTCCCTCCTCGCCAATG-3' Rv 5'-GGCGGGGACAGGCGAGCATC**AG**-3'	PCR/RFLP (*Mnl *I)	AA: 206 AG: 206,180,26 GG: 180,26	Modified from [19]
rs2010963	Fw 5'-CCGACGGCTTGGGGAGATTG**CTC**-3' Rv 5'-CGGCGGTCACCCCCAAAAG**CAG**-3'	PCR/RFLP (*BsmF *1)	GG:157, 40 GC: 197,157,40 CC: 197	Modified from [3]
rs25648	----	Real time PCR Taqman assay	----	hCV791476

*Nucleotides in bold letters in primer sequences indicate primer modifications included in this study. †Original references of primer sequences and protocols.

The evaluation of the rs25648 *VEGF *variant was performed using a predesigned 5'-nuclease assay with TaqMan MGB probes (Applied Biosystems: Foster City, CA). All reactions were performed in 10 μL of volume and 10 ng of genomic DNA. Standard PCR cycling conditions were used and the allelic discrimination was made using the StepOne RT-PCR system with the StepOne v2.0 software (Applied Biosystems).

All the SNPs were validated by direct sequencing of PCR products obtained from four patients using BigDye Terminator v3.1 Cycle Sequencing Kit (Applied Biosystems), and analyzed in an Avant 3100 Genetic Analyzer. To ensure reliability of the results, duplicate samples and known genotyped samples were included in the analysis as quality controls.

### Data Analysis

General variables of the study groups such as maternal age, gestational age, and number of pregnancies, were compared using the Student's *t *test. In the control group, SNPs genotypes were tested for departures from Hardy-Weinberg equilibrium by exact test. To compare known risk factors, allele, and genotype frequencies between the study groups, a chi-square test was performed. Linkage disequilibrium statistics and haplotypes analysis, including haplotypes frequency estimation, as well as an analysis of association between haplotypes and PE, were made using SNPStats software http://bioinfo.iconcologia.net/index.php?module=Snpstats. The Odds Ratio (OR) values were calculated for each allele, genotype, and haplotype. For the adjustment of gestational age, logistic regression analysis was used to compare allele and genotype frequencies between PE and the control group. Study power was calculated at the end of the study using Quanto software v1.2.4 [20]. For each SNP we considered its minor allele frequency as reported in the Mexican HapMap project. Depending on the SNP, in the analysis an effect of size 2 or greater was established.

The level of significance was set at *P *< 0.05. Statistical analysis was performed using Sigma Plot software v11.

## Results

One hundred and sixty four unrelated pregnant women (86 PE cases and 78 normotensive controls) were included in the study. The comparison among the general variables of the study population (Table 2) showed that gestational age was the only variable associated to PE (*P *= 0.028). We did not find an association between known risk factors and PE (*P *> 0.05). Genotype frequencies for all the SNPs included in the study were in Hardy-Weinberg equilibrium in the controls (*P *> 0.05). Table 3 displays the genotypes and minor allele frequencies for each SNP in cases and controls. The proportions for the minor allele for rs699947, rs1570360, rs2010963, and rs25648 *VEGF *SNPs were 0.33, 0.2, 0.39, and 0.17 in controls, and 0.39, 0.23, 0.41, and 0.15 in cases, respectively (Table 3). We did not observe significant differences between individual genotypes or allele frequencies of the studied *VEGF *SNPs in the cases and control groups (*P *values > 0.05).

**Table 2:** Demographic variables and risk factors of the study population.

Characteristic	Controls (n = 78)	PE cases (n = 86)	*P *value
Maternal age, mean (range) years	25 (17-36)	27 (15-38)	0.067
Gestational age, median (range) weeks	32 (16-38)	34 (17-37)	0.028*
Number of pregnancies, median (range)	2 (1-6)	2 (1-5)	0.614
Family history of PE, n (%)	5 (6.4)	7 (8.1)	0.337
Family history of type 2 Diabetes mellitus, n (%)	24 (30.76)	35 (40.7)	0.784
Family history of arterial hypertension, n (%)	31 (39.74)	37 (43.02)	0.409
Primipaternity, n (%)	24 (30.76)	27 (28.13)	0.531
Use of barrier contraceptive method, n (%)	29 (37.18)	28 (33.55)	0.392
Nulliparous, n (%)	28 (35.89)	35 (40.69)	0.995

**Table 3:** Comparison of allele and genotype frequencies between study groups.

dbSNP ID	Genotype and minor allele	Controls (n = 78)	PE Cases (n = 86)	OR (CI 95%)	*P *value
rs699947	CC	35 (0.45)	29 (0.34)	0.75 (0.27-2.08)	0.31
	CA	34 (0.44)	47 (0.55)		
	AA	9 (0.11)	10 (0.11)		
	Allele A	52 (0.33)	67 (0.39)	0.78 (0.49-1.23)	0.34
rs1570360	GG	49 (0.63)	51 (0.59)	0.94 (0.49-1.80)	0.57
	GA	27 (0.35)	30 (0.35)		
	AA	2 (0.02)	5 (0.06)		
	Allele A	31 (0.2)	40 (0.23)	0.82 (0.48-1.38)	0.54
rs2010963	GG	26 (0.33)	28 (0.33)	1.03 (0.52-2.03)	0.79
	GC	43 (0.55)	45 (0.52)		
	CC	9 (0.12)	13 (0.15)		
	Allele C	61 (0.39)	71 (0.41)	0.91 (0.58-1.42)	0.77
rs25648	CC	55 (0.70)	62 (0.72)	0.98 (0.49-1.98)	0.53
	CT	20 (0.26)	23 (0.27)		
	TT	3 (0.04)	1 (0.01)		
	Allele T	26 (0.17)	25 (0.15)	1.17 (0.64-2.13)	0.70

In a statistical approximation, an association between genotype/allele frequencies and severity of the disease was not found (table S1 of Additional file 1). To test the association between PE onset and *VEGF *genotype variants, the group of severe PE patients was classified in early and late PE, with a cut off at 35 GW. Again, no association between *VEGF *genotypes and PE onset was found (*P *values > 0.05; data not shown).

To study the association between PE and possible combinations of the *VEGF *variants, we performed a haplotype analysis on for the four *VEGF *SNPs. A linkage disequilibrium (LD) test, showed a LD association between the rs699947, rs1570360, rs2010963, and rs25648 *VEGF *variants (D' > 0.87, range: 0.882 to 0.998 *P *< 0.001). The data for D'statistic of each pairwise of SNPs and its p values are shown in table S2 of Additional file 2. A comparison between haplotypes frequencies of *VEGF *SNPs and their association with PE is summarized in Table 4. Five major combinations between *VEGF *SNPs studied were determined; four of them with frequencies above 10% (C-G-C-C, C-G-G-C, A-A-G-C, and A-G-G-T for rs699947, rs1570360, rs2010963, and rs25648 *VEGF *SNPs, respectively) and one of them, A-G-G-C, with a frequency of 1.5%.

**Table 4:** Analysis of haplotypes frequencies and their association with PE.

	SNP						
				
Haplotype*	rs699947	rs1570360	rs2010963	rs25648	Freq	OR (95% CI)	*P *value
1	C	G	C	C	0.3851	1	-
2	C	G	G	C	0.2308	1.31 (0.72-2.39)	0.38
3	A	A	G	C	0.1953	0.72 (0.37-1.37)	0.31
4	A	G	G	T	0.1358	0.92 (0.44-1.96)	0.84
5	A	G	G	C	0.015	1.63 (0.33-8.13)	0.55
							
*Global haplotype association p value*							0.12

* The SNP order which defined the VEGF haplotype structure was rs699947, rs1570360, rs2010963, and rs25648, respectively.

The *P *values for individual and global haplotype score tests (Table 4) did not indicate a significant difference in *VEGF *haplotype frequency profiles between cases and controls (*P *> 0.05).

Considering the sample size of 164, the study power was calculated in a range of 45.19% to 65.96% to detect an effect of size 2 or greater depending on the SNP considered.

## Discussion

Genetic predisposition is a well-documented risk factors for PE [21,22]; however, although several genes have been postulated as genetic susceptibility markers of the disease [21-24], there is not a recognized hereditary profile to explain the association [21]. Considering differences in genetic predisposition between populations and the important role of *VEGF *gene in the angiogenesis during the pregnancy, we conducted a case-control study, to test the association between the rs699947, rs1570360, rs2010963, and rs25648 *VEGF *genetic variants and PE. Consistent with the lack of association between -2578C/A (rs699947) *VEGF *genotypic/allelic frequencies in women with severe PE reported by Papazoglou D *et al *(2004) in Greece [13], we did not find an association between genotype/allele frequencies of the studied SNPs and PE or with severity of the disease (*P *> 0.05). However, our results contrasted with those reported by Sandrim VC *et al*. (2009) for Caucasic Brazilian women, who reported a positive association between -2578C/A (but not for -1154G/A) genotypes and PE [12]. Banyasz I *et al *(2006) suggested that carriers of the *VEGF*+405G (rs2010963) allele in an Hungarian population have a decreased susceptibility to PE and while carriers of the *VEGF*-2578A (rs699947) allele could be associated with an accelerated development of the disease in nulliparous pregnant women with severe PE [3]. In our study, carriers of the *VEGF*+405G (rs2010963) allele did not have a decreased susceptibility to PE and no association was found between allele/genotypes frequencies of the studied *VEGF *variants (including *VEGF*-2578 C/A) and PE onset. Discrepancies of *VEGF *allele/genotype frequencies and their association with PE, severity, or onset of disease, may be explained in part by the ethnic variability between populations and by differences in selection criteria of the studied groups. For example, the lack of consensus in the controls selection, which has included post-menopausal women with a history of at least two uncomplicated pregnancies, nulliparous pregnant women, women with uncomplicated pregnancies, or normotensive pregnant women, makes difficult the comparison between studies and may include some bias in the association reports.

PE susceptibility related to *VEGF *haplotypes has been considered in a very few studies; however, none of them has genotyped all the variants included in this work. We did not find differences in the identified haplotypes profiles between cases and controls. In contrast with our results, Sandrim VC *et al*. (2009) suggested a protective effect for the -2578C, -1154G and -634C haplotype (associated with higher *VEGF *gene expression) against the development of PE [12]. In our study, we did not include the -634G/C variant; however, although we performed a statistical analyses in which only the -2578C/A, -1154G/A SNPs were considered for this analysis, the lack of association between *VEGF *haplotypes frequencies and PE was consistent. Finally, some considerations should be highlighted: One of the major limitations of this work was the small sample size because it influenced the study power (calculated in a range of 45.19% to 65.96% depending on the SNP studied). In this same sense, the limited sample size affects the association analyses performed in sub-classified groups, as PE severity or PE onset, since sub-classifications reduced the numbers of study subjects and could introduce some bias in the statistical analysis. In our, study the nulliparous state was not considered as selection criteria or was subclassified for additional comparisons; however, this PE risk factor showed no difference between groups. Our control group included normotensive women, but their "current healthy pregnant state" does not discard the future presence of PE pregnancies.

In Mexico, there are no data about genetic susceptibility for PE associated to *VEGF *variants. Our study is the first protocol that evaluated the possible association between *VEGF *genotypes/haplotypes and PE, and the results obtained in the study suggest the presence of additional genetic variants different from rs699947, rs1570360, rs2010963, and rs25648 *VEGF *as markers for the genetic susceptibility to PE.

## Conclusion

In our study, there was no evidence of an association between allele, genotype or haplotype frequencies and PE, its severity or onset of the disease.

## Competing interests

The authors declare that they have no competing interests. The authors are responsible for the content and writing of the research paper.

## Authors' contributions

IGV and CCDLR carried out the samples processing and the molecular genetic studies, IGV drafted the manuscript. RCF and VMG participated in the design of the study and in the coordination of patient recruitment. JERM, EAGM, and EML participated in data acquisition, analysis and interpretation, and reviewed the manuscript. ARM and ROL were involved in the design of the study and in the preparation of the manuscript. MLMF conceived the study, participated in its design and coordination, performed the statistical analysis, and helped to draft the manuscript. All authors read and approved the final manuscript.

## Pre-publication history

The pre-publication history for this paper can be accessed here:

http://www.biomedcentral.com/1471-2393/11/35/prepub

## Supplementary Material

Additional file 1 **Comparison of alleles, genotypes, and haplotypes frequencies between study groups, according to disease severity**. Click here for file

Additional file 2 **Linkage Disequilibrium Analysis**. D' statistics and *P *values for each parwise of *VEGF *variants included in the study. Click here for file
