# Supplementary material for: No association between polymorphisms/haplotypes of the vascular endothelial growth factor gene and preeclampsia
Source: BMC Pregnancy Childbirth. 2011 May 16;11:35. doi: 10.1186/1471-2393-11-35 (PMC3112063; doi:10.1186/1471-2393-11-35)
Supplement: Additional file 1 — Comparison of alleles, genotypes, and haplotypes frequencies between study groups, according to disease severity. [file 1471-2393-11-35-S1.PDF]

Additional file 1.

Table S1. Comparison of alleles, genotypes, and haplotypes frequencies between study groups, according to their severity.

ID	Variant or combination	Controls (n=78)	Mild PE (n=51)	Severe PE (n=35)	*P* value
rs699947	CC	35 (0.45)	16 (0.31)	13(0.37)	0.363
	CA	34 (0.44)	26 (0.51)	21 (0.6)	
	AA	9 (0.11)	9 (0.18)	1 (0.03)	
	Allele A	52 (0.33)	44 (0.43)	23 (0.33)	0.2
rs1570360	GG	49 (0.63)	29 (0.57)	22 (0.63)	0.629
	GA	27 (0.35)	17 (0.33)	13 (0.37)	
	AA	2 (0.02)	5 (0.1)	(0.0)	
	Allele A	31 (0.2)	27 (0.26)	13 (0.19)	0.667
rs2010963	GG	26 (0.33)	21 (0.41)	5 (0.14)	0.405
	GC	43 (0.55)	22 (0.43)	25 (0.71)	
	CC	9 (0.12)	8 (0.16)	5 (0.14)	
	Allele C	61 (0.39)	38 (0.37)	35 (0.5)	0.2
rs25648	CC	55 (0.70)	35 (0.69)	27 (0.77)	0.688
	CT	20 (0.26)	15 (0.29)	8 (0.23)	
	TT	3 (0.04)	1 (0.2)	0 (0.0)	
	Allele T	26 (0.17)	17 (0.17)	8 (0.11)	0.677
*Haplotype 1					
rs699947	C	0.3715	0.3482	0.5	0.330
rs1570360	G				
rs2010963	C				
rs25648	C				
*Haplotype 2					
rs699947	C	0.2512	0.2204	0.1714	0.594
rs1570360	G				
rs2010963	G				
rs25648	C				
*Haplotype 3					
rs699947	A	0.165	0.2404	0.1857	0.595
rs1570360	A				
rs2010963	G				
rs25648	C				
*Haplotype 4					
rs699947	A	0.1309	0.1667	0.1143	0.656
rs1570360	G				
rs2010963	G				
rs25648	T				

*Haplotype 5					
rs699947<br>rs1570360<br>rs2010963<br>rs25648	A<br>G<br>G<br>C	0.0202	----	0.0286	1

* The SNP order which defined the VEGF haplotype structure was rs699947, rs1570360, rs2010963, and rs25648 respectively.
