# Supplementary material for: No association between polymorphisms/haplotypes of the vascular endothelial growth factor gene and preeclampsia
Source: BMC Pregnancy Childbirth. 2011 May 16;11:35. doi: 10.1186/1471-2393-11-35 (PMC3112063; doi:10.1186/1471-2393-11-35)
Supplement: Additional file 2 — Linkage Disequilibrium Analysis. D' statistics and P values for each parwise of VEGF variants included in the study. [file 1471-2393-11-35-S2.PDF]

Additional file 2.

Table S2. Linkage Disequilibrium Analysis. D' statistics and *P* values of each pairwise of VEGF variants included in the study.

**SNP**	rs699947	rs1570360	rs2010963	rs25648
rs699947	---	D´=0.920<br>*P* <0.0001	D´=0.913<br>*P* <0.0001	D´=0.919<br>*P* <0.0001
rs1570360	---	---	D´=0.882<br>*P* <0.0001	D´=0.998<br>*P* <0.0001
rs2010963	---	---	---	D´=0.891<br>*P* <0.0001
rs25648	---	---	---	---
